# The more you get, the more you give: Positive cascading effects shape the evolutionary potential of prenatal maternal investment

**DOI:** 10.1002/evl3.125

**Published:** 2019-07-02

**Authors:** Joel L. Pick, Erik Postma, Barbara Tschirren

**Affiliations:** ^1^ Department of Evolutionary Biology and Environmental Studies University of Zurich Winterthurerstrasse 190 8057 Zurich Switzerland; ^2^ Institute of Evolutionary Biology School of Biological Sciences, University of Edinburgh Edinburgh EH9 3JT United Kingdom; ^3^ Centre for Ecology and Conservation University of Exeter Penryn TR10 9FE United Kingdom

**Keywords:** Body size, cascading maternal effects, egg size, indirect genetic effects, response to selection

## Abstract

Maternal effects are prevalent in nature and significantly contribute to variation in phenotypic trait expression. However, little attention has been paid to the factors shaping variation in the traits mediating these effects (maternal effectors). Specific maternal effectors are often not identified, and typically they are assumed to be inherited in an additive genetic and autosomal manner. Given that these effectors can cause long‐lasting effects on offspring phenotype, it is likely that they may also affect themselves in the next generation. Although the existence of such cascading maternal effects has been discussed and modeled, empirical examples of such effects are rare, let alone quantitative estimates of their strength and evolutionary consequences. Here, we demonstrate that the investment a mother makes in her eggs positively affects the egg investment of her daughters. Through reciprocally crossing artificially selected lines for divergent prenatal maternal investment in Japanese quail (*Coturnix japonica*), we demonstrate that the size of eggs daughters lay resembles the egg size of their maternal line significantly more than that of their paternal line, highlighting that egg size is in part maternally inherited. Correspondingly, we find that variation in the daughters' egg size is in part determined by maternal identity, in addition to substantial additive genetic effects. Furthermore, this maternal variance in offspring egg size is fully explained by maternal egg size, demonstrating the presence of a positive cascading effect of maternal egg size on offspring egg size. Finally, we use an evolutionary model to quantify the consequences of covariance between cascading maternal and additive genetic effects for both maternal effector and offspring body mass evolution. Our study demonstrates that by amplifying the amount of variation available for selection to act on, positive cascading maternal effects can significantly enhance the evolutionary potential of maternal effectors and the offspring traits that they affect.

**Impact Summary**

As well as passing on genes, a mother shapes her offsprings phenotype by influencing the environment they experience early in life. Such maternal effects are ubiquitous in nature and are recognized for their impact on phenotypic trait expression. However, whether the traits causing these maternal effects also affect their own expression in subsequent generations (cascading maternal effects) has seldom been considered and the evolutionary implications of such feedback loops are not well understood. By extending quantitative genetic techniques and applying these to reciprocal crosses of lines of Japanese quail artificially selected for divergent prenatal maternal investment, we first establish the presence of non‐genetic, positive cascading maternal effects in maternal investment; the investment a mother makes in her eggs positively affects the egg investment of her daughters, over and above the effects of genes that a mother passes to her daughters. Using evolutionary modeling, we further demonstrate that this association between additive genetic and positive cascading maternal effects leads to an amplification effect, accelerating the evolutionary potential of both maternal investment and any other traits in offspring (e.g., body size) affected by this maternal investment. Our findings highlight the long‐term consequences of the care experienced by a female during the first stages of life on her ability to care for her own offspring, and the importance of taking such effects into account when attempting to predict evolutionary change in natural populations.

Mothers shape their offsprings' phenotype not only through the genes they pass on to them, but also by influencing the developmental environment their offspring experience early in life (Mousseau and Fox 1998). Both theoretical and empirical work has shown that such maternal effects on offspring phenotype are an important driver of the evolutionary dynamics of a trait (Falconer 1965; Kirkpatrick and Lande 1989; Wolf et al. 1998; Räsänen and Kruuk 2007; McGlothlin and Galloway 2014). From an evolutionary perspective, it is important to establish whether the maternal traits mediating these maternal effects (i.e., the maternal effectors) have a genetic basis, as this allows maternal effectors to respond to selection acting on the offspring traits that they affect (Wolf et al. 1998; Räsänen and Kruuk 2007; McAdam et al. 2014). A genetic basis thus enables maternal effectors to evolve alongside the offspring trait and, depending on the direction of the maternal effect, magnify or constrain the response of the offspring trait to selection (Kirkpatrick and Lande 1989; Galloway et al. 2009). Despite a large body of work focusing on how maternal effects influence the evolution of offspring characters, the maternal effectors themselves have received much less attention. Specific maternal effectors are often not identified and typically a simple, additive genetic inheritance pattern is assumed (Räsänen and Kruuk 2007) (cf. maternal genetic effects). Yet, if we assume that maternal effects can shape offspring phenotypes, then there is no good reason for a priori excluding a role of maternal effects in shaping variation in the maternal effectors themselves. Intriguingly, maternal effectors may even affect their “own” expression in subsequent generations, a phenomenon known as a “cascading” maternal effect (McGlothlin and Galloway 2014).

Cascading maternal effects may represent an important form of non‐genetic inheritance (Danchin et al. 2011), with interesting evolutionary dynamics. Offspring from larger litters, for example, typically grow more slowly (Falconer 1965; Schluter and Gustafsson 1993; McAdam et al. 2002; Wilson et al. 2005b; Ramakers et al. 2018) and reach a smaller adult size, which in turn results in smaller litters when these offspring reproduce themselves (Falconer 1965; Schluter and Gustafsson 1993; Jarrett et al. 2017; Ramakers et al. 2018). Therefore, despite litter size having a heritable basis and being under positive directional selection, the maternal environment it provides hinders its own response to selection. Given the capacity of such “negative” cascading effects to constrain a trait's response to selection, and thereby contribute to evolutionary stasis, their evolutionary significance is well appreciated (Janssen et al. 1988; Donohue 1999; Galloway et al. 2009). Examples of “positive” cascading effects, on the other hand, are scarce and largely descriptive.

Perhaps the best known example of a positive cascading effect is provided by maternal grooming behavior in rats (*Rattus norvegicus*). Female rats that are cross‐fostered between lines selected for divergent licking and grooming behavior, exhibit the licking and grooming behavior they have experienced as pups from their foster mother, rather than that of the line they originate from, when caring for their own young. In other words, licking and grooming behavior is non‐genetically maternally inherited (Francis et al. 1999). Similar patterns have been observed with aggressive behaviors in humans (Doumas et al. 1994), primates (Maestripieri 2005), and birds (Müller et al. 2011), whereby individuals who have experienced violence as juveniles are more likely to be violent toward their own offspring (known as the “Cycle of Violence”; Silver et al. 1969). Yet, although the mechanisms underlying the non‐genetic transmission of aggressive and maternal behaviors in some of these systems are now well understood, and the role of epigenetics in particular (Weaver et al. 2004; Champagne 2008; Curley and Champagne 2016), the evolutionary consequences of such positive cascading effects remain largely unexplored.

As additive genetic and positive cascading maternal effects are always positively correlated, positive cascading effects are predicted to magnify additive genetic effects (i.e., a daughter that has received a high level of investment herself invests more in her offspring than expected from her genes or the early life conditions she experienced alone; Fig. 1). The positive covariance between the two effects will therefore amplify the amount of phenotypic variation that is available for selection to act on and so increases the potential for a trait to respond to selection (we will refer to this covariance as the “amplification effect,” see also Eq. 1 in methods). Furthermore, whereas negative cascading effects are typically mediated by traits directly associated with maternal fitness (e.g., fecundity), positive cascading effects are associated with parental care, and so only have an indirect effect on maternal fitness through their effect on offspring fitness (Hadfield 2012), introducing additional complexity into their evolutionary dynamics.

**Figure 1:**
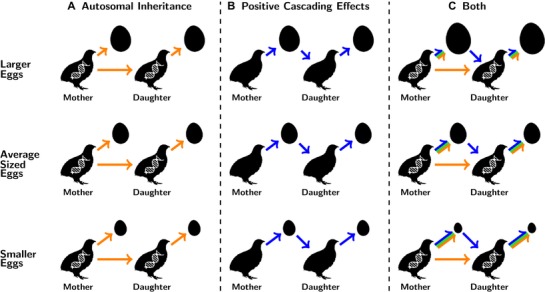
Inheritance patterns of maternal investment. A resemblance in egg investment between mothers and daughters can be due to A) additive genetic effects (orange) or B) non‐genetic, positive cascading maternal effects (blue). The joint contribution of additive genetic and positive cascading maternal effects (C) act to amplify each other, resulting in an additional amplification effect (green). Under this scenario, females investing heavily in their offspring, have daughters who investment even more in their own offspring than expected by either force alone, and visa versa.

Intergenerational effects do not only arise postnatally but also during the prenatal period. The estimation of such prenatal effects has, however, been hampered by the fact that they are not easily disentangled from additive genetic effects (Krist and Remeš 2004; Tschirren and Postma 2010; Pick, Ebneter, et al. 2016). Consequently, few studies have considered the long‐term effects of differential prenatal investment, and even fewer the effect of the prenatal environment on the future reproductive performance of the offspring (Krist 2011). In oviparous species, egg size is a key mediator of prenatal maternal effects (Bernardo 1996; Sogard 1997; Fox and Czesak 2000; Krist 2011), with strong positive effects on offspring phenotype, and offspring size in particular (Krist 2011; Pick, Ebneter, et al. 2016), a trait under strong directional selection (Rollinson and Rowe 2015). Given its long‐lasting effects and high heritability across taxa (Christians 2002), egg size presents an ideal model to quantify the occurrence of positive cascading maternal effects and their impact upon evolutionary dynamics. To this end, we here use reciprocal crosses between artificial selection lines for divergent prenatal maternal investment in Japanese quail (*Coturnix japonica*) (Pick, Ebneter, et al. 2016; Pick, Hutter, et al. 2016). We demonstrate that in addition to additive genetic, autosomal inheritance, prenatal maternal investment is also maternally inherited. Furthermore, by extending established quantitative genetic techniques, we show that prenatal maternal investment affects the prenatal maternal investment of the next generation. Finally, using an evolutionary model (Kirkpatrick and Lande 1989), we demonstrate how the simultaneous action of cascading maternal and additive genetic effects amplifies the evolutionary potential of both the maternal effector and the offspring trait that it affects.

## Results and Discussion

### MATERNAL INHERITANCE OF PRENATAL MATERNAL INVESTMENT

To test for the maternal inheritance of prenatal maternal investment, we reciprocally crossed birds from selection lines for divergent maternal egg investment (Pick, Hutter, et al. 2016; Pick, Ebneter, et al. 2016) within a breeding design in which both males and females were mated to two different partners, creating a mixture of full and half sibling offspring. Examining the egg size of the resulting F1 hybrids enabled us to distinguish between maternal and autosomal inheritance (Reznick 1981), as the hybrids have a similar intermediate autosomal genotype, but a different maternal background (i.e., either high or low investment). Maternal inheritance therefore manifests itself as the egg size of hybrids resembling the egg size of their maternal line significantly more than the egg size of their paternal line.

**Figure 2:**
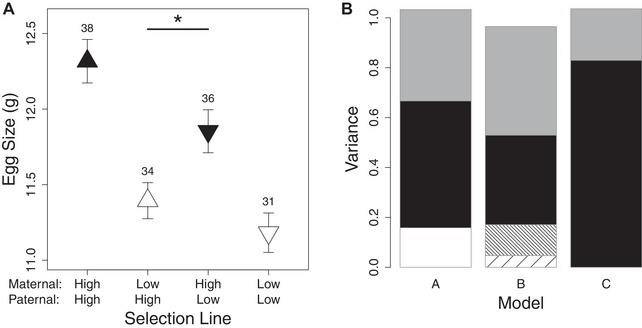
Evidence for cascading maternal effects. A) Egg sizes of pure‐bred and hybrid daughters from reciprocal crosses of the high and low maternal egg investment lines. Means ± SE and sample sizes of within‐pair means are shown. Colour represents the maternal line (black – High, white – Low) and symbol the paternal line (triangles – High, inverted triangles – Low). B) Variance components of egg size estimated using 3 animal models. Model A estimated additive genetic variance (*V_A_*, black) and total maternal variance (*V_M_*, white). Model B included maternal egg size as a covariate, which completely explained *V_M_*. The variance due directly to egg size (i.e. the positive cascading effect; $V_{M_{p}}$, coarse upward hatching) and covariance between the additive genetic and cascading effects (i.e. the amplification effect; *2CO* $V_{{A,M}_{p}}$, fine downward hatching), were not directly estimated in the models, but were derived from equations 6 and 16. Model C estimated only *V_A_*. In all models the residual variance is shown in grey. The total variance is lower in B because adding maternal egg size as a covariate also reduces VA (See Methods).

We found that the egg size of F1 females was significantly influenced by both the selection line of their mother ($\chi^{2}=29.19$, P<0.001) and their father ($\chi^{2}=7.65$, P=0.006). Yet the maternal line effect was significantly larger than the paternal line effect (z=2.332, P=0.010). In other words, hybrid females with a mother from the high egg investment line and a father from the low egg investment line laid significantly larger eggs than females with a mother from the low egg investment line and a father from the high egg investment line (Fig. 2A). This provides evidence for the partial maternal inheritance of egg size, over and above additive genetic autosomal effects inherited from both parents. There was no evidence of hybrid vigor (maternal x paternal line: $\chi^{2}=1.70$, P=0.192, Fig. 2A), and no differences between line replicates ($\chi^{2}=0.16$, P=0.693).

Our half‐sibling breeding design further allowed us to decompose the contribution of additive genetic and maternal effects to variation in egg size. Consistent with the analysis of the selection lines, the estimation of additive genetic variance ($\hat{V_{A}}$) and maternal variance ($\hat{V_{M}}$) using an animal model approach (model A) revealed a high heritability (*h* ^2^) of egg size (estimate ± SE: 0.508 ± 0.250, Fig. 2B; Table S1), alongside substantial maternal variance (*m* ^2^ = 0.158 ± 0.112, Fig. 2B, Table S1), although the latter was estimated with a large degree of error. Evidence for maternal inheritance of egg size has previously been found in wild bird populations (Larsson and Forslund 1992; Potti 1999; Budden and Beissinger 2005), alongside varied evidence from poultry (Hutt and Bozivich 1946; Sheridan and Randall 1977; Moritsu et al. 1997; Chang et al. 2009, see also Fox 1994). However, these studies were unable to identify the pathways by which such maternal resemblance is mediated or to disentangle cascading maternal effects from other forms of maternal inheritance (Pick, Ebneter, et al. 2016).

### POSITIVE CASCADING EFFECTS ON EGG SIZE

In order to test if the observed maternal effect on daughter's egg size is attributable to the mothers egg size, we included maternal egg size as a covariate in the model outlined above (model B; Fig. 2B, Table S1). In this model, $\hat{V_{M}}$ was reduced to 0, indicating that the increased resemblance among daughters sharing the same mother was explained entirely by maternal egg size (McAdam et al. 2014). Correspondingly, there was a significant positive effect of maternal egg size on offspring egg size ($b=0.473\pm 0.060,F_{1,118.1}=61.45,P<0.001$), providing evidence for a positive cascading effect of maternal egg investment on egg investment of the next generation. This conclusion is corroborated by an albumen removal experiment in chickens (*Gallus gallus*), in which daughters originating from eggs that had had albumen (the main source of protein for developing embryos) removed, subsequently produced smaller eggs with less albumen as adults (Willems et al. 2013, see also Mizuma and Hashima 1961, but note that such experiments are inherently problematic; discussed in Pick, Ebneter, et al. 2016). As of yet the mechanism(s) by which this nongenetic inheritance of maternal investment occurs remains to be elucidated. Work in rats has shown that maternal care can trigger epigenetic changes in the offspring, which in turn influence the future care strategy of the offspring (Champagne 2008; Curley and Champagne 2016). Hence, this presents a possible mechanism by which nongenetic transmission may occur in other systems, including our quail model.

Typically, the estimate of the effect of maternal phenotype on offspring phenotype is considered to represent the strength of the maternal effect (or maternal effect coefficient *m*; McAdam et al. 2014). However, as the maternal and offspring trait are the same, this estimate (*b*) is composed of both additive genetic and cascading maternal effects (see Eqn. 5). Furthermore, $\hat{V_{M}}$ from model A includes both the variance in offspring egg size due to the cascading maternal effect ($V_{M_{p}}$), as well as the positive covariance between additive genetic and cascading maternal effects (${\mathrm{COV}}_{A,M_{p}}$; i.e., the amplification effect; Eqn. 3). In order to disentangle these different components, and so estimate the strength of the positive cascading maternal effect (*p*) and the degree to which it amplifies the additive genetic effect, we extended the approach of Falconer (1965, see Methods). Using $\hat{V_{M}}$ from models A and B and *b* from model B, we found a positive cascading maternal effect (*p* = 0.217; Eqn. 16) that explained a small proportion of variation in offspring egg size (*p* ^2^ = 0.047; Fig. 2B). Although this nongenetic effect of maternal egg size on offspring egg size was comparably small, because of the substantial additive genetic variance in egg size, the amplification effect ($2{\mathrm{COV}}_{A,M_{p}}$) contributed to approximately 12% of the variation in egg size (${\mathrm{COV}}_{A,M_{p}}=0.062$; Fig. 2B, Eqn. 6). Therefore, the cascading maternal effect of maternal egg size on offspring egg size acted to substantially amplify the additive genetic effect.

### CONSEQUENCES OF POSITIVE CASCADING MATERNAL EFFECTS ON THE EVOLUTIONARY DYNAMICS OF EGG SIZE AND BODY SIZE

Selection for increased maternal investment occurs indirectly, via its impact on offspring fitness, rather than directly via the mother's fitness. Therefore, the evolution of such maternal effectors cannot be considered in isolation from the traits that they affect (Hadfield 2012). Egg size in particular is known to have a strong positive effect on juvenile body size (Krist 2011; Pick, Ebneter, et al. 2016), which is under strong directional selection (Kingsolver and Pfennig 2004; Rollinson and Rowe 2015). We therefore used the Kirkpatrick–Lande (K–L) model (Kirkpatrick and Lande 1989) to quantify the evolutionary consequences of the positive cascading maternal effect observed in our study on the rate of evolutionary change in egg size and juvenile body size. The K–L model quantifies how interacting traits (such as egg size and juvenile body size) respond to selection, and has both strong theoretical and empirical support (Hadfield 2012; McGlothlin and Galloway 2014). We used estimates presented in this study, alongside estimates from a previous study to parameterize the model (Pick, Ebneter, et al. 2016, see Methods). In the Supporting Information, we also jointly estimate these parameters for juvenile size and egg size in a bivariate animal model. The results do not differ from those presented here (Fig. S1), with the exception that the estimated genetic correlation between the two traits is non‐zero (albeit with a large confidence intervals that overlap zero; Table S2). The general conclusions relating to the impact of cascading maternal effects remain unaltered, with this positive genetic correlation generally increasing the effects reported below (Fig. S2).

**Figure 3:**
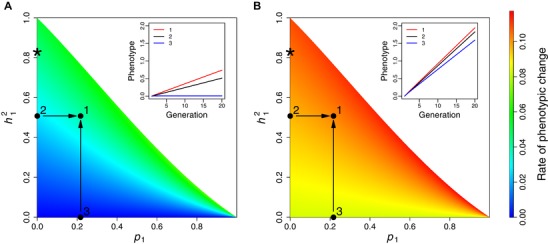
Asymptotic rate of evolution of a) maternal (egg size) and b) offspring (body size) traits, over varying heritability and cascading effects in the maternal effector (*h^2^_1_* and *p_1_*, respectively). Points represent evolutionary rates from different combinations of estimates of cascading maternal and additive genetic effects from this study; (1) with additive genetic effects and cascading maternal effects (2) with additive genetic effects only and (3) with cascading maternal effects only. Inserts to the figures show the predicted phenotypic change of the two traits under the three different scenarios and constant selection. The asterisk show the evolutionary rate predicted when the maternal effector is assumed to have only additive genetic effects, as in a maternal genetic effect (model C).

Comparing K–L models parameterized with the same additive genetic effects (as estimated in model A), but either including or not including a positive cascading effect of maternal egg size on offspring egg size (as estimated here), revealed that a positive cascading effect substantially increases the asymptotic rate of evolution of this maternal effector by 43% (Fig. 3A, points 1 and 2). The cascading effect also increased the rate of evolution of juvenile body size, although to a smaller degree (6%; Fig. 3B, points 1 and 2). On the other hand, comparing K–L models parameterized with the same positive cascading effect of maternal egg size on offspring egg size, but either including or not including additive genetic effects, revealed that in the absence of additive genetic effects on egg size, the evolutionary rate of juvenile body size decreased by 18% (Fig. 3B, points 1 and 3), while the rate of evolution of egg size reduced to 0 (Fig. 3A, points 1 and 3). Although cascading maternal effects clearly have the potential to substantially alter the response to selection, an additive genetic component underlying the maternal effector is essential for these cascading effects to influence the evolutionary potential of the maternal effectors and the offspring traits that they affect. Evidence of a phenotypic cascading effect alone is therefore not sufficient to infer how (or whether) these effects may influence evolutionary dynamics.

### BIASES IN THE ESTIMATE OF EVOLUTIONARY RATES WHEN NOT CONSIDERING CASCADING MATERNAL EFFECTS

Typically, maternal effectors are not individually identified, but grouped into a “maternal performance” trait, which is assumed to be inherited in a purely autosomal fashion (i.e., when modeled as a maternal genetic effect in a variance component approach; Wilson et al. 2005a; Hadfield 2012; McAdam et al. 2014). To demonstrate the effect that the violation of this assumption has on estimates of evolutionary rates, we analyzed the line cross experiment with an animal model that only estimated additive genetic variance in egg size ($\hat{V_{A}}$; model C) and used the resulting estimates to parameterize the K–L model. As expected (Kruuk 2004; Kruuk and Hadfield 2007), the absence of the maternal variance term (in model A) substantially upwardly biased the heritability estimate of egg size (0.829 ± 0.197, Fig. 2B, Table S1). Consequently, the evolutionary rates were overestimated by 14% for egg size and 2.6% for juvenile body size (Fig. 3A,B). This bias would increase with an increasing contribution of the cascading effect to the overall heritable (*sensu lato*) component of the maternal effector (i.e., higher *p*_1_ and lower $h_{1}^{2}$ in Fig. 3), to the extent that with purely cascading effects the maternal effector would not evolve, while being predicted to, and so would have no effect on the evolutionary rate of juvenile body size. The presence of cascading effects, when not explicitly modeled, thus leads to a consistent upward bias in the estimation of maternal genetic effects (as shown by the difference in *h* ^2^ between models A and C) (Kruuk and Hadfield 2007), and so an upward bias in the prediction of the evolutionary rate of both maternal effectors and the offspring traits that they affect (Fig. 3A,B; see also McGlothlin and Galloway 2014). The accuracy of predictions of a trait's evolutionary potential therefore crucially depends on both the identification of maternal effectors, and on a correct understanding of their inheritance patterns.

### CONCLUSIONS

In conclusion, our study provides empirical evidence for positive cascading maternal effects, which, by amplifying the amount of variation available for selection to act on, affect the evolutionary potential of both prenatal maternal investment and juvenile body size. Evolutionary models show that such positive cascading maternal effects only influence evolutionary dynamics in the presence of additive genetic effects. Our results therefore demonstrate that both additive genetic effects and cascading maternal effects have to be estimated simultaneously to obtain unbiased estimates of evolutionary rates. Furthermore, our results highlight the importance of taking a trait‐based approach to understanding maternal effectors, and thereby their potential to shape phenotypic evolution.

## Methods

### SELECTION FOR DIVERGENT MATERNAL INVESTMENT

We used Japanese quail from established, replicated selection lines for divergent maternal investment (i.e., high egg investment and low egg investment). Information on the selection regime, the line crosses, and on general husbandry procedures are presented in Pick, Ebneter, et al. (2016); Pick, Hutter, et al. (2016). In brief, we selected for high and low maternal egg investment, measured as egg size corrected for female body size, with each selection line replicated twice. After three generations of directional selection, the divergent lines differed in absolute egg size by 1.2 SD. The lines were then reciprocally crossed to create F1 hybrids. To this end, a total of 80 females and 80 males (20 individuals per sex and line replicate) were each bred twice, once with an individual of their own line, and once with an individual of the other line, resulting in both pure‐bred and hybrid half‐sib F1 offspring (Pick, Ebneter, et al. 2016). After reaching sexual maturity, F1 females (*N* = 297 daughters, from the 139 pairings, of 78 fathers and 77 mothers, that resulted in any adult daughters) were bred with a random male to determine their mean egg size (to the nearest 0.01 g; *N* = 1–27 eggs per female).

### STATISTICAL ANALYSES

We used a number of complementary statistical approaches to quantify the long‐term consequences of prenatal maternal investment on the egg investment of the next generation:

#### Maternal versus paternal line effects

We modeled the effect of maternal and paternal line (high or low investment), their interaction, and line replicate on F1 female egg size using a linear mixed effects model. Paternal ID, maternal ID, and the interaction between the two were included as random effects to account for the non‐independence of offspring from the same parents. In addition to estimating the effect of the maternal and paternal line on the daughters egg investment, we also tested specifically whether the maternal line effect was significantly larger than the paternal line effect (one‐sided *z*‐test) following Hothorn et al. (2008).

In the absence of any effect of maternal egg size on daughter egg size over and above that of the genes for egg size passed on by parents to their daughters, we expect the effect of maternal and paternal line on offspring egg size to be identical and therefore both types of F1 hybrids to have egg sizes that are intermediate to the two pure‐bred groups. Alternatively, if there is an additional effect of maternal egg investment on the egg investment of the next generation (i.e., a positive cascading maternal effect), we would expect the maternal line effect to be significantly stronger than the paternal line effect. This would manifest itself as hybrid females whose mother originated from the high investment line laying significantly larger eggs than hybrid females whose mother originated from the low investment line. However, as discussed in Pick, Ebneter, et al. (2016), a stronger maternal than paternal line effect demonstrates the presence of maternal inheritance (*sensu lato*), rather than positive cascading maternal effects specifically. In other words, from the comparison of the selection lines alone, we cannot rule out other sources of maternal resemblance, such as mitochondrial or W‐linked inheritance. We present further analyses aimed at quantifying their relative roles below. Finally, an interaction between maternal and paternal line would be indicative of hybrid vigor.

Egg size was *z*‐transformed to have a mean of 0 and a standard deviation of 1. We performed stepwise backwards elimination of nonsignificant terms. Maternal and paternal line terms and all random effects were always retained in the models. The statistical significance of fixed effects was determined by comparing models, fitted using maximum likelihood, with and without the variable of interest using a likelihood ratio test. The degrees of freedom for all tests was 1. Analyses were performed in the R statistical framework (version 3.0.3) (R Core Team 2014) using the packages lme4 (version 1.1‐6) (Bates et al. 2015) for model fitting and comparison, and multcomp (version 1.4‐1) (Hothorn et al. 2008) for within‐model comparison of maternal and paternal line effects.

#### Maternal effects and egg size

Variance in offspring egg size ($V_{P}$) can be decomposed into

(1) $$V_{P}=V_{A}+V_{M_{p}}+2{\mathrm{COV}}_{A,M_{p}}+V_{M_{r}}+V_{R}$$

where $V_{A}$ is the additive genetic variance, $V_{M_{p}}$ is the variance attributable to the effect of maternal egg size on egg size in the next generation over and above the additive genetic variance (mediated by the cascading effect *p*; i.e., the effect of a mothers egg investment on the daughters egg investment), ${\mathrm{COV}}_{A,M_{p}}$ is the covariance between additive genetic and cascading effects, $V_{M_{r}}$ is the variance attributable to the mother not explained by the cascading effect, and $V_{R}$ is the residual variance. The latter includes variance due to random environmental effects and any effects of dominance and epistasis (Falconer 1965). Crucially, because maternal egg size is a function of a females additive genetic value for egg size, which she passes on to her daughters, a positive cascading effect of maternal egg size on offspring egg size (i.e., p>0) will introduce a positive covariance between offspring breeding value and maternal effect value, i.e., ${\mathrm{COV}}_{A,M_{p}}>0$, giving rise to the amplification effect.

We used a hybrid variance component/trait‐based model approach (McAdam et al. 2014) in which we used nested “animal models” to quantify the contribution of maternal egg size to the total maternal variance component for offspring egg size ($V_{M}$). In short, an “animal model” is a type of mixed effects model that estimates $V_{A}$ and other components of variance by utilizing the relatedness among all individuals in a pedigree (Henderson 1988; Kruuk 2004), in this case among parents, full‐ and half‐sib offspring. For these models, we used the data from our half sibling breeding design; we used the phenotypes of the F1 offspring and pedigree consisting of the F1 offspring and their parents. Although we have phenotypic and pedigree information for more generations of the selection lines, our selection procedure led to extremely high assortative mating and produced only full‐sib families, meaning that additive genetic and cascading maternal effects cannot be distinguished in previous generations.

Model A included a random additive genetic (“animal”) and a maternal identity effect, enabling the separation of the role of additive genetic and maternal effect variance in shaping variation in offspring egg size. This model decomposes $V_{P}$ to

(2) $$\hat{V_{P}}=\hat{V_{A}}+\hat{V_{M}}+\hat{V_{R}}$$

were $\hat{V_{M}}$ is the estimate of the maternal variance (i.e., the variance attributable to maternal identity). From equation 1, it follows that $\hat{V_{M}}$ as estimated in this model captures variation from different maternal sources:

(3) $$\hat{V_{M}}=V_{M_{p}}+2{\mathrm{COV}}_{A,M_{p}}+V_{M_{r}}$$

This in contrast to the estimate of $V_{A}$ ($\hat{V_{A}}$), which is not confounded with any other source of variation (i.e., is unbiased).

Model B differs from model A in that it includes maternal egg size (mean size of all incubated eggs from each mother) as an additional co‐variate. Because the relationship between maternal egg size and offspring egg size is part genetic and part maternal in origin, we would expect both $\hat{V_{M}}$ and $\hat{V_{A}}$ to decrease from model A to model B. The size of the decrease in $\hat{V_{M}}$ between the two models is a measure of the contribution of maternal egg size to $\hat{V_{M}}$, and thus $\hat{V_{M}}$ reduces to $\hat{V_{M_{r}}}$ (McAdam et al. 2014). Therefore, if egg size is the sole maternal trait influencing offspring egg size, $\hat{V_{M}}$ will reduce to zero. Unlike the maternal effect, where the maternal phenotype may represent the trait causing the effect, the maternal phenotype does not directly represent the maternal genotype. Therefore, the proportional reduction in $\hat{V_{A}}$ as a result of the inclusion of maternal egg size is related to the proportion of the variance in maternal breeding values that is explained by the maternal phenotype or, in other words, the correlation between maternal phenotype and maternal breeding value, i.e., *h* ^2^. However, as half of the genetic variance in the offspring trait is attributable to variation in paternal rather than maternal breeding values, the proportional reduction in $\hat{V_{A}}$ is equal to $\frac{h^{2}}{2}$. Note this reduction may be dependent on our use of mean offspring and mean maternal egg size.

Finally, model C included a random additive genetic effect only, providing an estimate of the additive genetic variance ($\hat{V_{A}}$) assuming no other sources of resemblance among full‐ and half‐sibs. It is well known that by not estimating $V_{M}$ when maternal effects exist, $V_{A}$ will be overestimated (Kruuk and Hadfield 2007). The estimate of $V_{A}$ provided by this model allows us to demonstrate the effect that not accounting for maternal effects on the maternal effector has on the estimation of the selection response (see below).

Because both offspring and maternal egg size were *z*‐transformed to have a mean of 0 and a standard deviation of 1, and animal models A and C included a fixed intercept only, $\hat{V_{A}}$ is equivalent to *h* ^2^ (narrow‐sense heritability) and $\hat{V_{M}}$ to *m* ^2^ (proportion of variance due to maternal identity). All animal models were run in ASReml‐R (version 3.0; Gilmour et al. 2009). The significance of fixed effects was estimated on the basis of conditional Wald F statistics.

#### Decomposing the effect of maternal egg size on offspring egg size

Previous work has shown that *p* can be estimated from covariances between additive genetic and maternal genetic effects, using phenotypic data on both parents and offspring over at least three generations (Galloway et al. 2009; McGlothlin and Brodie 2009). However, as discussed above, here we use phenotypic data from our F1 hybrids only, and so our dataset does not allow for the cascading maternal effect to be estimated in this way. As an alternative approach, we extended the methods of Falconer (1965) (outlined below), which allows for the analysis of more restricted datasets. Future work should seek to compare the two methods.

In most implementations of the hybrid model B, the traits measured in mother and offspring are different, and assuming the absence of a genetic correlation between the maternal and the offspring trait, the slope of the offspring phenotype on the maternal phenotype (*b*) represents the maternal effect. However, because in our case both traits are highly genetically correlated (indeed, they are the same trait), the estimated slope is a function of both the strength of the maternal effect and the heritability of the trait. Here, we extend the work of Falconer (1965), enabling us to estimate the strength of the cascading maternal effect (*p*; the partial regression coefficient of offspring egg size on maternal egg size, after accounting for additive genetic effects).

Following Falconer (1965), the covariance between maternal egg size ($P^{\prime }$) and offspring egg size (*P*) can be decomposed into

(4) $${\mathrm{COV}}_{P^{\prime },P}=\frac{V_{A}}{2-p}+pV_{P}$$

If both offspring and maternal egg size are *z*‐transformed to have a standard deviation of 1 (i.e., $V_{P}$ = 1 and $V_{A}$ = *h* ^2^), Equation 4 becomes

(5) $$b=\frac{h^{2}}{2-p}+p$$

where *b* is the slope of maternal egg size on offspring egg size.

Furthermore, again following Falconer (1965), the covariance between an offspring's breeding value (*A*) and its cascading maternal effect value ($M_{p}$) is equal to

(6) $${\mathrm{COV}}_{A,M_{p}}=\frac{pV_{A}}{2-p}$$

Hence, we can rewrite equation 3 as

(7) $$\hat{V_{M}}=V_{M_{p}}+\frac{2pV_{A}}{2-p}+V_{M_{r}}$$

which, when traits are standardized to have a phenotypic variance of 1, gives

(8) $${\hat{m}}^{2}=p^{2}+\frac{2ph^{2}}{2-p}+m_{r}^{2}$$

where ${\hat{m}}^{2}$ is the estimated proportion of variance in the offspring phenotype explained by maternal identity, *p* ^2^ is the proportion of variance that is attributable to cascading maternal effects, and $m_{r}^{2}$ is the proportion of the phenotypic variance attributable to other aspects of the mother.

To obtain *p*, equation 5 can be rearranged to

(9) $$b-p=\frac{h^{2}}{2-p}$$

and 8 can be rearranged to

(10) $$\frac{{\hat{m}}^{2}-m_{r}^{2}-p^{2}}{2p}=\frac{h^{2}}{2-p}$$

These can now be combined to give

(11) $$\frac{{\hat{m}}^{2}-m_{r}^{2}-p^{2}}{2p}=b-p$$

(12) $$p^{2}-2bp+{\hat{m}}^{2}-m_{r}^{2}=0$$

We can solve this for *p* using the quadratic formula:

(13) $$x=\frac{-c\pm \sqrt{c^{2}-4ad}}{2a}$$

where

(14) $$ax^{2}+cx+d=0$$

When applied to equation 12, a=1, c=−2b and $d={\hat{m}}^{2}-m_{r}^{2}$. Hence,

(15) $$p=b\pm \sqrt[{}]{b^{2}-{\hat{m}}^{2}+m_{r}^{2}}$$

Assuming the cascading maternal effect is positive (i.e., p>0), then

(16) $$p=b-\sqrt[{}]{b^{2}-{\hat{m}}^{2}+m_{r}^{2}}$$

From this it follows that *p* (and *p* ^2^) can be estimated using the estimates of *b* and $V_{M_{r}}$ obtained from model B, and using the estimate of $V_{M}$ obtained from model A.

#### Evolutionary dynamics of egg size and juvenile body size

Direct selection on offspring traits affected by maternal investment results in indirect selection for increased maternal investment (Hadfield 2012). In order to understand the evolutionary dynamics of a maternal effector, we therefore have to take into account its role in shaping trait expression in the next generation. For example, in addition to the effects of maternal egg size on offspring egg size explored above, egg size also has a strong effect on other aspects of offspring phenotype, and in particular on juvenile body size (Krist 2011; Pick, Ebneter, et al. 2016), which is under strong directional selection (Kingsolver and Pfennig 2004; Rollinson and Rowe 2015). To understand the effect that different inheritance patterns of the maternal effector (i.e., egg size) have on the evolutionary rate of both egg size and juvenile body size, we therefore used the K–L model (Kirkpatrick and Lande 1989, eq. 7) (see Hadfield 2012; McGlothlin and Galloway 2014 for a discussion of the utility of this model) to estimate the asymptotic rate of evolution of maternal egg size $\Delta \bar{z}\left(\infty \right)$:

(17) $$\Delta \bar{z}\left(\infty \right)={\left(I-M\right)}^{-1}C_{\mathrm{az}}\beta$$

In this two‐trait model, M is the maternal effect matrix (composed of maternal effect coefficients),

(18) $$M=\left[\begin{array}{cc} p_{1} & \;0\\ m_{1,2} & \;0 \end{array}\right]$$

where subscripts 1 and 2 refer to egg size and juvenile body size, respectively, and *m*_1, 2_ refers to the effect of trait 1 (egg size) on trait 2 (juvenile size). Furthermore, **I** is an identity matrix

(19) $$I=\left[\begin{array}{cc} 1 & \;0\\ 0 & \;1 \end{array}\right]$$

and $C_{\mathrm{az}}$ is a matrix of covariances between breeding values and phenotypes, calculated as

(20) $$C_{az}=G{\left(I-{}^{1}{/}_{2}M^{T}\right)}^{-1}$$

which in the absence of any maternal effects is equal to the additive genetic variance–covariance matrix **G**

(21) $$G=\left[\begin{array}{cc} \;\;V_{A_{1}} & \;{\mathrm{COV}}_{A_{2},A_{1}}\\ {\mathrm{COV}}_{A_{1},A_{2}} & \;\;V_{A_{2}} \end{array}\right]$$

Finally, β is a vector of selection gradients

(22) $$\beta =\left[\begin{array}{c} \beta_{1}\\ \beta_{2} \end{array}\right]$$

The model was parameterized using estimates for egg size obtained from the analyses above (heritability $h_{1}^{2}$ and cascading effect *p*_1_). As our measure of juvenile size, we used body mass at two weeks post‐hatching, which is the age at which juveniles become independent (Orcutt and Orcutt 1976; Launay et al. 1993). Across taxa, selection on juvenile size is much stronger than on adult size (Rollinson and Rowe 2015) and in many bird species size at independence has been shown to strongly predict survival and recruitment (Tinbergen and Boerlijst 1990; Both et al. 1999). Selection is, therefore, likely to be strongest at this point. We used estimates from Pick, Ebneter, et al. (2016) for the heritability of juvenile size ($h_{2}^{2}=0.378$) and the maternal effect of egg size on juvenile size (*m*_1, 2_ = 0.483). No evidence for a genetic correlation between egg size and juvenile size (${\mathrm{COV}}_{A_{1},A_{2}}$) was found in this previous study, so this was set to 0. Note that these estimates for juvenile size and egg size were obtained in separate analyses, and it is possible that the point estimates may differ if estimated together. We therefore also estimated these parameters for juvenile and egg size jointly in a bivariate animal model, which we present in the Supporting Information. The results do not differ from those presented here (Fig. S1), with the exception that the estimated genetic correlation is nonzero (albeit with a large confidence intervals that overlap zero; Table S2). As our emphasis here is on the impact of positive cascading maternal effects on evolutionary potential, we here assume the true genetic correlation between the two traits is zero, but we explore the potential consequences of a nonzero genetic correlation in the Supporting Information. We have no direct measure of selection on juvenile body size in our captive population, but a recent study showed that the median selection gradient on juvenile size (β_2_) across a large number of studies was 0.22 (Rollinson and Rowe 2015). We therefore used this value as an estimate of the strength of selection acting on the juvenile body size and assumed there to be no direct selection on egg size (i.e., β_1_ = 0, but see Cheverud 1984; Hadfield 2012; Thomson et al. 2017).

Initially, we parameterized the model with all possible values of both *p*_1_ and $h_{1}^{2}$ to demonstrate how both the heritability and the strength of cascading effects in the maternal effector (egg size) influence the rate of evolution in both traits. Because in all models the phenotypic variance $V_{P}$ for both egg size and juvenile body size was 1, $h_{1}^{2}+p_{1}^{2}+\frac{2h_{1}^{2}p_{1}}{2-p_{1}}\geq 1$ (i.e., as $V\left(x+y\right)=V_{x}+V_{y}+2{\mathrm{COV}}_{x,y}$; see also Eq. 6). From these predictions, we extracted the predicted evolutionary rates of egg size and juvenile body size for our estimates of both additive genetic and cascading effects (point 1 in Fig. 3; using estimates from animal models A and B). We then compared these predictions to those from a model where $h_{1}^{2}$ was the same but *p*_1_ was set to 0, to demonstrate the impact of the cascading maternal effects we estimated here (point 2 in Fig. 3). We also compared these with a model that was parametrized with our estimate of *p*_1_, but with $h_{1}^{2}$ set to 0, to demonstrate the impact of the cascading maternal effects occurring in the absence of additive genetic effects (point 3 in Fig. 3). Finally, we parameterized the K–L model using estimates from animal model C (i.e., assuming that the maternal effector showed autosomal inheritance only) to demonstrate the impact that not accounting for cascading effects in the maternal trait has on predictions of evolutionary rates.

Associate Editor: A. Charmantier

## Supporting information


**Table S1**: Model estimates (±SE) from three animal models of offspring egg size.
**Table S2**: Model estimates from two bivariate models of egg size and juvenile size (± SE).
**Figure S1**: Comparison of variance components estimated from univariate (uni) and multivariate (multi) models of juvenile size and egg size.
**Figure S2**: Asymptotic rate of evolution of a) maternal (egg size) and b) offspring (body size) traits, over varying heritability and cascading effects in the maternal effector ($h_{M}^{2}$ and *p*, respectively).
