# Supplementary material for: The more you get, the more you give: Positive cascading effects shape the evolutionary potential of prenatal maternal investment
Source: Evol Lett. 2019 Jul 2;3(4):412–23. doi: 10.1002/evl3.125 (PMC6675147; doi:10.1002/evl3.125)
Supplement: Supplementary file 1 — Table S1: Model estimates (±SE) from three animal models of offspring egg size. Table S2: Model estimates from two bivariate models of egg size and juvenile size (± SE). Figure S1: Comparison of variance components estimated from univariate (uni) and multivariate (multi) models of juvenile size and egg size. Figure S2: Asymptotic rate of evolution of a) maternal (egg size) and b) offspring (body size) traits, over varying heritability and cascading effects in the maternal effector (hM2 and p, respectively). [file EVL3-3-412-s001.pdf]

The more you get, the more you give: Positive cascading effects shape the evolutionary potential of prenatal maternal investment

Supplementary Material

Joel L. Pick, Erik Postma & Barbara Tschirren

Joint estimation of parameters for juvenile and egg size

The parameter estimates for juvenile size and egg size used in the K-L models were estimated separately, and it is possible that the point estimates would be different if estimated together. For example, if only one of the traits was affected by maternal egg size alongside an environmental covariance between juvenile size and egg size, univariate analyses of the two traits could lead to the appearance of maternal variance attributable to egg size in both traits. In order to test this, we complemented the univariate models presented both in the main text and in Pick *et al.* (2016), with additive genetic and maternal variances for juvenile and egg size jointly in a bivariate animal model. Similar to the structure of the univariate models, hatch day (day 17 or day 18 of incubation) was included as a fixed factor for juvenile size only (chicks hatching later are smaller, an effect which disappears by adulthood; Pick *et al.*, 2016), with no fixed effects for egg size (Model D; the bivariate version of model A). In a second model, mean maternal egg size was included as a covariate for both traits (Model E; the bivariate version of model B). Both models included a random additive genetic ('animal') and a maternal identity effect for both traits, and the covariance between them. A common rearing environment effect was also included for juvenile size only (see Pick *et al.*, 2016). As the maternal variances for both traits were effectively reduced to 0 in the second model, the maternal covariance term was excluded to aid model convergence. For comparison with previous models, both traits were z-transformed. Models were run in ASReml-R (version 3.0 Gilmour *et al.*, 2009).

Given the results of the univariate analyses, we may expect several outcomes. First, we may find maternal variance in both traits similar to those estimated in the univariate analyses, with a strong maternal covariance between the two, demonstrating that the same maternal effector (egg size) is underlying the maternal variance in the two traits, supporting the assumptions made in the main text. Alternatively, we may find non-zero maternal variances in both traits, but no covariance, suggesting both traits are shaped by different maternal effectors. This scenario is unlikely, as including maternal egg size as a covariate reduces the maternal variance to 0 in both traits (see main text and Pick *et al.* 2016). Finally, we may find non-zero maternal variance in only one trait, and a strong residual correlation between the two traits, leading to the two maternal effect coefficients being confounded when estimated in univariate analyses.

The variance components estimated in the bivariate model were extremely similar to those estimated in univariate analyses (Tables S1 and S2, Figure S1), with maternal variance

in both traits. There was a very strong maternal correlation between the two maternal effects (0.930 ± 0.312; Table S2) and little residual correlation (-0.075 ± 0.231; Table S2), suggesting that the same maternal effector is causing the maternal effect in both traits. Concordant with univariate analyses, the maternal variance reduced to 0 in both traits when maternal egg size was included as a covariate (Table S2). The multivariate analysis also gave very similar point estimates to the univariate analyses for maternal variance and maternal effect coefficients (Table S2, Figure S1). p in the multivariate model was estimated as 0.238 and COV_{A,M_p} as 0.069.

In a previous paper, the absence of a genetic correlation between egg and offspring size was inferred from the absence of a paternal line effect on juvenile size (Pick *et al.*, 2016). In our bivariate analysis this genetic correlation was estimated to be non-zero (0.368 ± 0.255), although the error on this estimate is large.

To explore the impact this genetic correlation may have on our conclusions relating to the evolutionary potential of egg size and juvenile size, we re-ran the set of K-L models described in the main text with a genetic correlation between the two traits of 0.368. As the genetic variance in egg size changed across the range of models (and phenotype variance was kept constant at 1), the genetic covariance was calculated as $COV_{A1,A2} = r_A\sqrt{h_1^2 h_2^2}$.

Generally this positive genetic correlation substantially increases the evolutionary potential of the maternal effector (Figure S2). The general conclusions relating to the impact of cascading maternal effects remain unaltered, however. The presence of cascading maternal effects alongside additive genetic effects acts to substantially increase the evolutionary potential of egg size. The evolutionary rate of egg size was 35% higher in a K-L model parameterised with a cascading effect than without one, and 10% higher for juvenile size (Figure S2, points 1 and 2). In the absence of genetic variation for egg size, there was no response of egg size to selection on juvenile size (Figure S2, point 3), and a decrease of 38% in the evolutionary rate of juvenile size. Interestingly, in the presence of a genetic correlation between the two traits, both traits evolve most rapidly in the presence of a very strong cascading effect and a low (but non-zero) heritability of the maternal effector (Figure S2).

Table S1: Model estimates (±SE) from three animal models of offspring egg size. Model A estimated additive genetic variance (V_A), total maternal variance (V_M) and residual variance (V_R). Model B additionally included maternal egg size as a covariate, which completely explained V_M. Model C estimated only V_A. Offspring and maternal egg size were z-transformed (mean=0, sd=1).

	Model		
	A	B	C
Fixed Effects			
Intercept	-0.017 ± 0.088	-0.001 ± 0.068	-0.017 ± 0.089
Maternal Egg Size	-	0.473 ± 0.060	-
Random Effects			
V_A	0.508 ± 0.250	0.358 ± 0.125	0.829 ± 0.197
V_M	0.158 ± 0.112	0.000 ± 0.000	-
V_R	0.367 ± 0.155	0.436 ± 0.104	0.207 ± 0.134

Table S2: Model estimates from two bivariate models of egg size and juvenile size (± SE). r is the correlation between the random effects of two traits. Both traits have been z-transformed (mean=0, sd=1).

	Trait		
	Egg	r	Juvenile
**Model D**			
Fixed Effects			
Intercept	-0.023 ± 0.089	-	0.013 ± 0.088
Hatching Day (18)	-	-	-0.610 ± 0.086
Maternal Egg Size	-	-	-
Random Effects			
V_A	0.510 ± 0.239	0.368 ± 0.255	0.400 ± 0.129
V_M	0.182 ± 0.114	0.930 ± 0.312	0.194 ± 0.073
V_E	-	-	0.060 ± 0.022
V_R	0.360 ± 0.148	-0.075 ± 0.231	0.371 ± 0.074
**Model E**			
Fixed Effects			
Intercept	-0.004 ± 0.068	-	0.048 ± 0.073
Hatching Day (18)	-	-	-0.610 ± 0.086
Maternal Egg Size	0.501 ± 0.064	-	0.431 ± 0.047
Random Effects			
V_A	0.362 ± 0.125	0.186 ± 0.185	0.383 ± 0.078
V_M	0	-	0
V_E	-	-	0.060 ± 0.022
V_R	0.434 ± 0.104	0.034 ± 0.142	0.381 ± 0.053
Phenotypic Mean	11.74		54.12
Phenotypic Variance	1.16		54.88

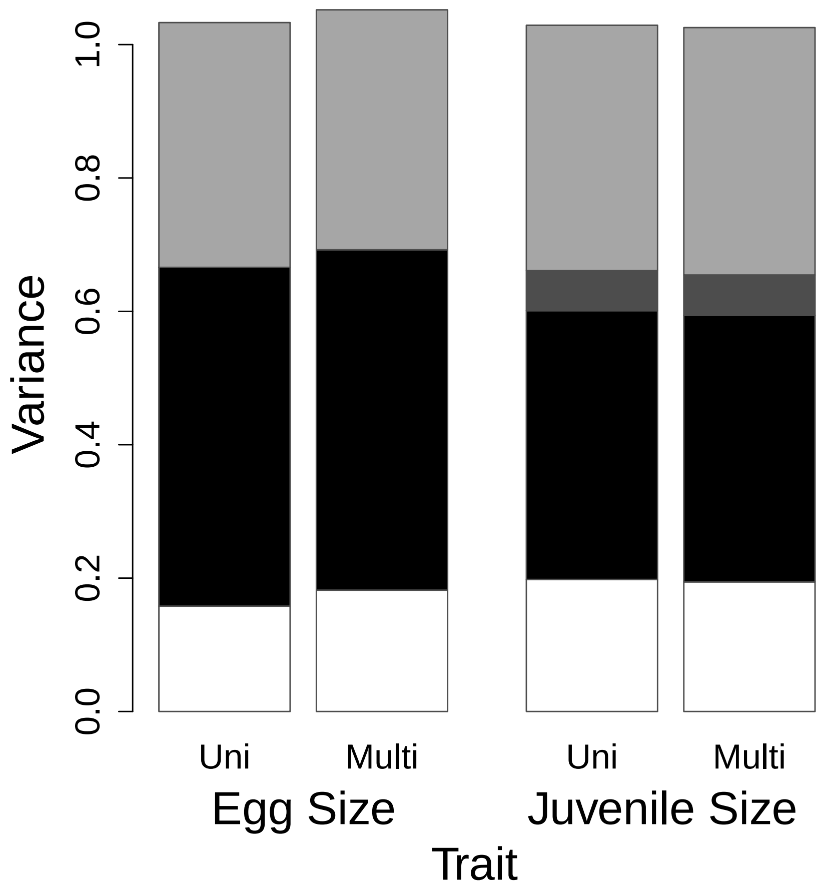

Figure S1: Comparison of variance components estimated from univariate (uni) and multivariate (multi) models of juvenile size and egg size. Additive genetic variance (V_A) is shown in black, maternal variance (V_M) in white and residual variance in light grey. The model for juvenile size also included a common rearing environment effect (dark grey).

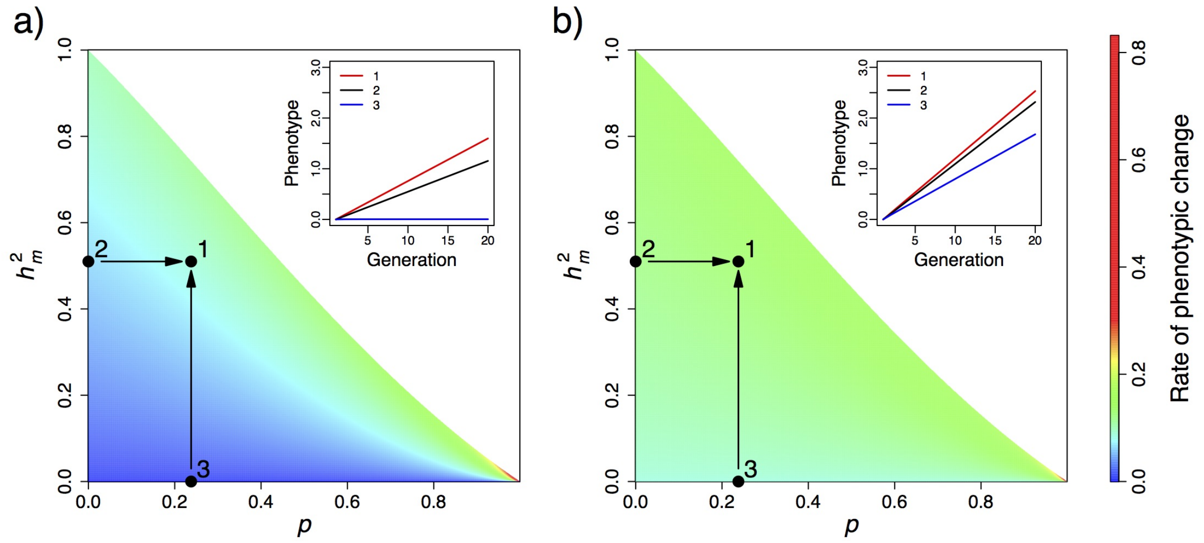

Figure S2: Asymptotic rate of evolution of a) maternal (egg size) and b) offspring (body size) traits, over varying heritability and cascading effects in the maternal effector (h^2_M and p, respectively). Points represent evolutionary rates from different combinations of estimates of cascading maternal and additive genetic effects from bivariate models; (1) with additive genetic effects and cascading maternal effects (2) with additive genetic effects only and (3) with cascading maternal effects only. Inserts to the figures show the predicted phenotypic change of the two traits under the three different scenarios and constant selection. Note the difference in the scale between this figure and figure 3 in the main text.

References

Gilmour, A., Gogel, B., Cullis, B. & Thompson, R. (2009). ASReml User Guide Release 3.0.

Pick, J.L., Ebneter, C., Hutter, P. & Tschirren, B. (2016). Disentangling genetic and prenatal maternal effects on offspring size and survival. *Am. Nat.* 188:628–639.
